# E-health Literacy and the Impact of Telehealth on Treatment Compliance and Accessibility

**DOI:** 10.7759/cureus.103718

**Published:** 2026-02-16

**Authors:** Nouran Abdellatif, Israa Omar Qais, Suleiman Rami Hussain, Hanadi Abbas AlJabal, Ahmad Alhimairi, Hussein Naji

**Affiliations:** 1 Public Health, Gulf Medical University, Ajman, ARE; 2 Cardiology, Canadian Specialist Hospital, Dubai, ARE; 3 Pediatric Surgery, Mohammed Bin Rashid University for Medical Sciences, Dubai, ARE; 4 Pediatric Surgery, Mediclinic Parkview Hospital, Dubai, ARE

**Keywords:** e-health literacy, healthcare accessibility, online consultation, telehealth, treatment compliance

## Abstract

Telehealth has become a transformative advancement in healthcare delivery, facilitating the remote provision of medical services and broadening healthcare accessibility. This study explores the relationship between e-health literacy, treatment compliance, and telehealth accessibility among users in the United Arab Emirates (UAE). Employing a cross-sectional design, 400 adults from diverse demographic backgrounds were surveyed to assess their telehealth knowledge, treatment adherence, and perceived access to care. Results show significant associations between individual components of e-health literacy, and self-reported treatment compliance and healthcare accessibility. Participants with greater e-health literacy demonstrated significantly higher compliance with remote treatment plans and reported enhanced access to healthcare, particularly among individuals encountering physical or logistical challenges. Of the 199 participants familiar with the definition of telehealth, 167 (83.9%) noted improved compliance with remote treatment plans, while 178 (89.4%) indicated enhanced healthcare access. These results emphasize the critical role of e-health literacy in optimizing telehealth effectiveness and suggest that initiatives aimed at enhancing e-health literacy may further promote positive telehealth outcomes.

## Introduction

Telehealth refers to the remote delivery of healthcare services using information and communication technologies, aimed at enhancing access to care and improving health outcomes [1]. It represents a significant advancement in healthcare, helping to overcome barriers such as geographical distance, time constraints, and physical limitations [2]. While the concept of telehealth has existed for decades, its utilization has surged in recent years, particularly following the global COVID-19 pandemic, catalyzing rapid integration into routine clinical care across the globe [3]. Even years after the peak of COVID-19, numerous healthcare departments continue to report sustained utilization of telehealth services, underscoring its enduring value in healthcare delivery [4]. Numerous studies have highlighted the potential benefits of telehealth, including improved access to healthcare, enhanced patient compliance, better treatment outcomes, and increased efficiency for healthcare providers [5]. Recent studies have also emphasized the significant role of utilizing telehealth to reduce barriers to healthcare access in rural and underserved areas [6]. 

However, the effectiveness of telehealth is not universally consistent across all studies. Various factors, including patient and provider familiarity with digital health platforms, influence its outcomes. One critical factor is e-health literacy, or the ability of individuals to seek, understand, and use digital health information effectively. The success of telehealth largely depends on the level of e-health literacy among both patients and healthcare professionals, which can significantly shape the outcomes of remote care [7].

While existing research has explored telehealth in the United Arab Emirates (UAE), there remains a notable gap in understanding how e-health literacy influences the effectiveness of telehealth, particularly in terms of patient compliance and accessibility to care. A previous study conducted in the UAE reported positive attitudes toward telehealth, with observed improvements in both patient compliance and healthcare accessibility [5]. However, there hasn't been a comprehensive study on the specific role of e-health literacy in determining these outcomes.

The primary objective of this study is to examine the association between e-health literacy and telehealth-related outcomes among adults in the UAE. Specifically, the study aims to explore the role of e-health literacy in shaping telehealth utilization, treatment compliance, and perceived healthcare accessibility by (i) comparing e-health literacy levels between individuals with and without prior telehealth use; (ii) assessing the relationship between e-health literacy and anticipated treatment compliance and perceived healthcare accessibility among individuals who have not previously used telehealth services; and (iii) descriptively evaluating telehealth experiences among individuals with prior telehealth use.

By addressing these objectives, the study seeks to contribute to the growing body of literature on digital health equity and to inform targeted interventions aimed at optimizing telehealth delivery in similar healthcare systems.

## Materials and methods

Study design and setting

This study employed a cross-sectional design to examine the relationship between e-health literacy and telehealth utilization, compliance, and perceived accessibility among adults in the UAE. The study was conducted at Thumbay University Hospital, Ajman, UAE, between April 15, 2022, and February 20, 2023. Participants were recruited using a convenience sampling method from adult patients attending outpatient clinics during the study period.

Study population

The study included adults aged 18 years and older of both genders and various nationalities residing in the UAE. Individuals who did not provide informed consent, those younger than 18 years of age, and non-residents of the UAE were excluded from the study.

Sample size

The sample size was calculated using the formula N = 4PQ/L², where N represents the required sample size, P is the estimated prevalence, Q = 1 − P, and L is the allowable margin of error. Based on this calculation and to reflect the demographic diversity of the UAE population, a target sample size of 400 participants was determined. To account for potential incomplete responses, an additional 40 participants were initially recruited; however, 400 fully completed questionnaires were included in the final analysis.

Study instrument

A structured, self-administered questionnaire was developed and distributed in English (Appendix 1). The questionnaire consisted of multiple sections assessing sociodemographic characteristics, knowledge and awareness of telehealth, prior telehealth utilization, and perceptions related to compliance and accessibility. Content validity was ensured through expert review, and a pilot study involving 10 participants was conducted to assess clarity and feasibility. Pilot participants were excluded from the final analysis.

Measures

E-health Literacy

E-health literacy was assessed using six knowledge-based items (Questions 7-12) evaluating participants’ awareness and understanding of telehealth concepts and applications. Responses were recorded on a four-point ordinal scale (“Don’t know,” “Not sure,” “Somewhat aware,” and “I am aware”) and coded numerically from 0 to 3, with higher scores indicating greater e-health literacy. A composite e-health literacy score was calculated as the mean of these six items [8].

Compliance and Perceived Accessibility Among Non-Users

Participants who reported no prior use of telehealth services were asked to complete a set of items assessing anticipated compliance and perceived accessibility if telehealth were to be used. This domain consisted of five items (Questions 31-35) addressing likelihood of treatment adherence, comfort with telehealth-based care, increased healthcare utilization, and perceived facilitation of access, including for individuals with physical or psychological barriers. Responses were recorded on a five-point Likert scale ranging from “Never” (0) to “Always” (4). A composite compliance and accessibility score for non-users was calculated as the mean of these items. These items were analyzed only among participants who had never used telehealth services.

Telehealth Experience Among Users

Participants who reported prior use of telehealth services completed a separate set of questions assessing telehealth experience, including method of telehealth delivery, source of telehealth services, convenience, communication quality, satisfaction, and self-reported compliance. These items (Questions 15-30) were analyzed descriptively and were not combined into a composite domain score; instead, responses were recorded on a 5-point Likert scale (0-4), “Strongly Disagree” (0) to “Strongly Agree, (4)” with higher scores indicating more positive perceptions of telehealth.

Reliability analysis

Internal consistency of the e-health literacy domain and the compliance and perceived accessibility domain for non-users was assessed using Cronbach’s alpha. The e-health literacy domain demonstrated excellent reliability (α = 0.97; 6 items). The compliance and perceived accessibility domain for non-users demonstrated good reliability (α = 0.87; 5 items)[9].

Data collection and statistical analysis

Questionnaires were distributed using a mixed approach, including online (Google Forms) and paper-based formats, to ensure accessibility for participants with varying levels of digital literacy. Responses were reviewed for completeness prior to analysis. Data were entered into Microsoft Excel and subsequently analyzed using the Statistical Package for the Social Sciences (SPSS) version 31.0.2.

Descriptive statistics, including frequencies, percentages, means, and standard deviations, were used to summarize participant characteristics and questionnaire responses. Independent samples t-tests were used to compare mean e-health literacy scores between telehealth users and non-users. Pearson’s correlation coefficients were used to assess associations between e-health literacy scores and the compliance and perceived accessibility scores among non-users. A two-tailed p-value of less than 0.05 was considered statistically significant.

Ethical approval

The study was approved by the Institutional Review Board of Gulf Medical University (IRB Ref. No. IRB/COM/STD/68/April-2022). All ethical guidelines were followed to ensure participant confidentiality, privacy, and anonymity. Written informed consent was obtained from all participants prior to participation.

## Results

This study comprised a total of 400 participants, of whom 225 (56.3%) were male. The study population was ethnically diverse, with 179 (44.8%) participants of Arab nationality and 178 (44.5%) of Asian nationality. Smaller proportions of participants were African (n = 10, 2.5%), European or North American (n = 21, 5.3%), or of other nationalities (n = 12, 3.0%).

Participants were distributed across different age groups. The largest proportion was aged 25-35 years (n = 128, 32.0%), followed by those aged 35-45 years (n = 109, 27.3%) and those older than 45 years (n = 99, 24.8%). Participants younger than 25 years comprised 64 people (16.0%) in the sample.

Regarding educational attainment, nearly half of the participants held an undergraduate degree (n = 190, 47.5%), followed by those with a high school diploma (n = 145, 36.3%). Smaller proportions had a master’s degree (n = 32, 8.0%), medical education (n = 15, 3.8%), no formal education (n = 14, 3.5%), or a doctoral degree (n = 4, 1.0%) (Table 1).

Among the 400 participants, 63 (15.8%) reported having previously used telehealth services, while 337 (84.3%) reported no prior telehealth use. The mean e-health literacy score was significantly higher among telehealth users compared to non-users (2.57 ± 0.74 vs. 1.36 ± 1.28, respectively).

An independent samples t-test demonstrated a statistically significant difference in e-health literacy scores between telehealth users and non-users (t = −10.43, df = 142.44, p < 0.001), indicating that participants with higher e-health literacy were more likely to have utilized telehealth services (Table 2).

**Table 1:** Frequency distribution of sociodemographic characteristics

Variable	Group	Number (n=400)	Percentage (%)
Age Group	<25	64	16.0
	25-35	128	32.0
	35-45	109	27.3
	>45	99	24.8
Gender	Female	175	43.8
	Male	225	56.3
Nationality	Asians	178	44.5
	Arabs	179	44.8
	Africans	10	2.5
	Europeans and North Americans	21	5.3
	Other nationalities	12	3.0
Education	No education complete	14	3.5
	High school diploma	145	36.3
	Undergraduate	190	47.5
	Masters	32	8.0
	PhD/Doctorate	4	1.0
	Medical Education	15	3.8
Marital status	Single	121	30.3
	Married	279	69.8
Employment	Unemployed	65	16.3
	Student	42	10.5
	Employed	293	73.3

**Table 2:** Comparison of e-health literacy scores between telehealth users and non-users Values are presented as mean ± SD. An independent-samples t-test was used. Higher e-health literacy scores indicate greater e-health literacy.

Variable	Non-users mean ± SD (n = 337)	Users (yes) mean ± SD (n = 63)	t (df)	p-value
E-Health Literacy Score	1.36 ± 1.28	2.57 ± 0.74	−10.43 (142.44)	< 0.001

Amongst participants who had not previously used telehealth services (n = 337), the association between e-health literacy and perceptions of compliance and accessibility was examined using Pearson’s correlation analysis. A composite compliance and accessibility score was calculated for non-users based on items assessing anticipated adherence to treatment plans and perceived accessibility of care through telehealth.

A moderate positive correlation was observed between e-health literacy scores and the combined compliance and accessibility score (r = 0.418, p < 0.001), indicating that higher e-health literacy scores were associated with higher compliance and accessibility scores among non-users (Table 3).

**Table 3:** Correlation between e-health literacy score and compliance, and accessibility score among non-users of telehealth Pearson’s correlation was used to assess the association between the e-health literacy score and the combined compliance and accessibility score among non-users of telehealth. Higher scores reflect greater e-health literacy and more favorable perceived compliance and accessibility toward telehealth.

Variable	Pearson’s r	p-value
Compliance & accessibility score	0.418	< 0.001

Participants who had previously used telehealth services (n = 63) reported generally positive experiences across multiple domains related to accessibility, convenience, communication, and overall satisfaction (Table 4).

**Table 4:** Descriptive statistics of telehealth experience among users (n = 63) Values are presented as mean ± SD. Responses were recorded on a 5-point Likert scale (0–4), with higher scores indicating more positive perceptions of telehealth.

Statement	Mean ± SD
Telehealth improved access to healthcare services	2.32 ± 0.64
Telehealth saved time and travel inconvenience	3.54 ± 0.62
Telehealth reduced waiting time for appointments	3.27 ± 0.90
Telehealth was easy and simple to use	3.22 ± 0.96
Telehealth met my healthcare needs	2.97 ± 1.03
Communication with the doctor was clear	3.25 ± 0.84
I was able to express my concerns clearly	3.08 ± 0.99
The telehealth session was convenient	3.24 ± 0.73
Scheduling a telehealth appointment was easier than a regular visit	3.00 ± 0.95
I was compliant with the treatment plan received through telehealth	3.02 ± 1.02
Overall telehealth experience was satisfactory	3.22 ± 0.83
I would recommend telehealth to others	3.19 ± 0.91

High mean scores were observed for statements indicating that telehealth saved time and reduced travel inconvenience (mean ± SD: 3.54 ± 0.62), reduced waiting time for appointments (3.27 ± 0.90), and was easy to use (3.22 ± 0.96). Communication during telehealth consultations was also rated favorably, with participants reporting clear communication with healthcare providers (3.25 ± 0.84) and the ability to express concerns effectively (3.08 ± 0.99).

Overall satisfaction with telehealth services was high (3.22 ± 0.83). Users also reported favorable compliance with treatment plans delivered through telehealth (3.02 ± 1.02) and a high likelihood of recommending telehealth to others (3.19 ± 0.91), reflecting positive user perceptions of telehealth-based care.

## Discussion

One of the primary objectives of this study was to evaluate the relationship between e-health literacy and its influence on telehealth utilization, patient compliance, and perceived accessibility to healthcare services among adults in the UAE. The findings demonstrate a statistically significant association between higher e-health literacy and both the use of telehealth services and more favorable perceptions of compliance and accessibility. These results highlight the importance of digital health knowledge in shaping patient engagement with telehealth, even in the absence of prior experience.

Participants who had previously utilized telehealth services demonstrated significantly higher e-health literacy scores compared to non-users. This suggests that individuals with greater understanding and awareness of telehealth concepts may be more likely to adopt telehealth as a mode of healthcare delivery. This finding is consistent with prior international studies reporting that higher levels of e-health literacy are associated with greater acceptance and utilization of telehealth services, as well as improved patient engagement in digital healthcare environments [10-18].

Importantly, the present study also examined the relationship between e-health literacy and perceptions of compliance and accessibility among participants who had not previously used telehealth services. A moderate positive correlation was observed between e-health literacy scores and anticipated compliance and accessibility, indicating that individuals with higher e-health literacy were more likely to report favorable expectations regarding treatment adherence and healthcare access through telehealth. By restricting this analysis to non-users, the study was able to assess baseline attitudes toward telehealth independent of prior exposure, reducing the potential influence of experience-related bias. These findings suggest that e-health literacy plays a critical role not only in telehealth adoption but also in shaping readiness and openness toward telehealth-based care.

In addition to these findings, descriptive analysis of participants who had previously used telehealth services revealed generally positive experiences across domains related to accessibility, convenience, communication, and overall satisfaction. Users reported that telehealth saved time, reduced travel burden, and facilitated access to care, while also enabling clear communication with healthcare providers and satisfactory treatment adherence. These observations are consistent with studies from the United States, Australia, and other healthcare systems, which have similarly reported high satisfaction, improved access, and enhanced convenience among telehealth users [13,15,19-25].

Collectively, the results of this study support the notion that telehealth can serve as a valuable tool for improving healthcare accessibility and patient compliance, particularly when supported by adequate levels of e-health literacy. Enhancing digital health literacy may, therefore, represent a key strategy for increasing telehealth adoption and optimizing its effectiveness within the UAE healthcare system. From a public health perspective, improving e-health literacy may represent a modifiable target to enhance telehealth utilization and effectiveness.

Several strengths and limitations should be considered when interpreting these findings. A key strength of this study is the inclusion of a diverse sample drawn from a multicultural population in the UAE, enhancing the relevance of the findings to similar healthcare settings. Additionally, the use of composite domain scores to assess e-health literacy and telehealth-related outcomes, along with expert review, pilot testing, and reliability assessment of the questionnaire, strengthens the methodological rigor and internal consistency of the study.

However, several limitations should also be acknowledged. First, the cross-sectional design precludes causal inference between e-health literacy and telehealth-related outcomes. Second, the reliance on self-reported questionnaire data may introduce reporting or recall bias. Additionally, although the study population was diverse in terms of nationality and educational background, the relatively small proportion of telehealth users and the single-center sampling approach may limit the generalizability of the findings, particularly to older adults, rural populations, or individuals with limited digital access. The limited number of participants above 45 years of age also restricted further age-based stratification, which may limit age-specific interpretations of the findings. Another limitation is that the study did not assess the direct or indirect costs associated with telehealth services, which may influence adoption and accessibility, particularly in low- and middle-income settings. Future studies using longitudinal designs and larger, more representative samples are warranted to further explore the causal pathways between e-health literacy and telehealth outcomes.

## Conclusions

This study highlights the significant role that e-health literacy plays in enhancing both treatment compliance and accessibility to healthcare services through telehealth, shedding light on the potential of telehealth services to build more efficient healthcare systems when implemented effectively. As telehealth continues to expand in the UAE and globally, it is essential to address gaps in e-health literacy to ensure that patients can fully benefit from the advantages that telehealth offers.

Furthermore, the establishment of targeted educational initiatives and public health strategies aimed at improving digital health skills and e-health literacy is essential for optimizing telehealth use, particularly among vulnerable populations. Future research should focus on identifying barriers to e-health literacy and evaluating the effectiveness of interventions designed to improve patient outcomes within digital healthcare environments.

## Appendices

Appendix 1 

**Table 5:** Appendix 1: Questionnaire

No.	Section	Question	Response Options
1	Sociodemographic	Age	Open response
2	Sociodemographic	Sex	Female / Male
3	Sociodemographic	Nationality	Open response
4	Sociodemographic	Educational level	A. High school / B. University / C. PhD / D. Masters / E. Medical / F. None
5	Sociodemographic	Marital status	Single / Married
6	Sociodemographic	Occupation	Open response
7	Telehealth Literacy	Telemedicine is the use of telecommunication to provide medical information and services	Don't know / Not sure / I am aware
8	Telehealth Literacy	Telemedicine provides healthcare where distance is a problem	Don't know / Not sure / I am aware
9	Telehealth Literacy	Face-to-face interaction with doctors is possible through telemedicine	Don't know / Not sure / I am aware
10	Telehealth Literacy	Patients’ drug management can be done via telemedicine	Don't know / Not sure / I am aware
11	Telehealth Literacy	Patient exams/investigations can be communicated via telemedicine	Don't know / Not sure / I am aware
12	Telehealth Literacy	Follow-up can be done through telemedicine	Don't know / Not sure / I am aware
13	Technology Use	Do you use online technology for education/meetings/appointments?	Yes / No
14	Technology Use	Have you previously had a telehealth appointment?	Yes (Answer Q15-28) / No (Skip to Q29)
15	Technology Use	Which method did you use to receive telehealth?	Email / Video meeting / Phone call / Text or web messaging
16	Technology Use	Source of telehealth appointment	Certified hospital / Gov source / Physician / Internet site / Holistic
17	Technology Use	Telehealth improved access to healthcare	Strongly Agree / Agree / Neutral / Disagree / Strongly Disagree
18	Technology Use	Telehealth saved you travel time	Strongly Agree / Agree / Neutral / Disagree / Strongly Disagree
19	Technology Use	Telehealth saved you time waiting for appointments	Strongly Agree / Agree / Neutral / Disagree / Strongly Disagree
20	Technology Use	Telehealth is easy and simple to use	Strongly Agree / Agree / Neutral / Disagree / Strongly Disagree
21	Technology Use	Telehealth meets your healthcare needs	Strongly Agree / Agree / Neutral / Disagree / Strongly Disagree
22	Technology Use	Clear conversation with doctor during telehealth	Strongly Agree / Agree / Neutral / Disagree / Strongly Disagree
23	Technology Use	Able to express feelings/concerns clearly	Strongly Agree / Agree / Neutral / Disagree / Strongly Disagree
24	Technology Use	Telehealth session was convenient	Strongly Agree / Agree / Neutral / Disagree / Strongly Disagree
25	Technology Use	Scheduling telehealth was easier than regular	Strongly Agree / Agree / Neutral / Disagree / Strongly Disagree
26	Technology Use	Compliant with treatment via telehealth	Strongly Agree / Agree / Neutral / Disagree / Strongly Disagree
27	Technology Use	Overall telehealth experience was satisfactory	Strongly Agree / Agree / Neutral / Disagree / Strongly Disagree
28	Technology Use	Would recommend telehealth to a friend	Strongly Agree / Agree / Neutral / Disagree / Strongly Disagree
29	Technology Use	Would use telehealth if available	Strongly Agree / Agree / Neutral / Disagree / Strongly Disagree
30	Technology Use	Telehealth is convenient for time/transport	Strongly Agree / Agree / Neutral / Disagree / Strongly Disagree
31	Compliance & Access	How likely are you to be compliant with a treatment plan?	Scale: Never (0) to Always (4)
32	Compliance & Access	Will you be more compliant if treated via telehealth?	Scale: Never (0) to Always (4)
33	Compliance & Access	Would you feel more comfortable with medications via telehealth?	Scale: Never (0) to Always (4)
34	Compliance & Access	Would you visit a physician more if telehealth were available?	Scale: Never (0) to Always (4)
35	Compliance & Access	If you have disability/anxiety, will telehealth help access care?	Scale: Never (0) to Always (4)
